# Performance Testing and Evaluation of Double-Layer Pervious Concrete Based on Recycled Aggregates

**DOI:** 10.3390/ma19061067

**Published:** 2026-03-11

**Authors:** Wencan Jiao, Zhengyang Peng, Bin Ma, Chunyu Dai, Bin Gong, Zhen Huang

**Affiliations:** 1 Guangxi Beibu Gulf Investment Group Co., Ltd., Nanning 530029, China; 2 Guangxi Xinfazhan Communication Group Co., Ltd., Nanning 530000, China; 3 School of Civil and Architectural Engineering, Guangxi University, Nanning 530004, China; 4 Guangxi Road Construction Engineering Group Co., Ltd., Nanning 530001, China

**Keywords:** recycled fine aggregates, pervious concrete, mix proportion optimization, paste-to-coarse aggregate ratio, ecological slope protection

## Abstract

A double-layer pervious concrete composite structure incorporating recycled fine aggregates derived from construction waste was developed to advance ecological slope protection performance. Single-factor experimental investigations on single-layer pervious concrete examined the effects of recycled fine aggregate replacement ratios (0–60%) and water–cement ratios (0.27–0.39) on material properties. The experimental results established 0.36 as the optimal water–cement ratio, while a 45% replacement ratio achieved an effective balance between permeability and compressive strength. Subsequently, parametric studies on double-layer composite concrete evaluated paste-to-coarse aggregate ratios ranging from 0.3 to 0.55. A paste-to-coarse aggregate ratio of 0.45 yielded optimal compressive strength while preserving favorable permeability characteristics, thereby achieving an effective balance between hydraulic and mechanical performance. Field tests of slope protection demonstrated that the double-layer configuration exhibited superior water retention capacity within the planting layer, while the fine particle layer effectively attenuated infiltration rates. Interlayer capillary mechanisms facilitated vertical moisture redistribution, ensuring equilibrated moisture distribution across soil strata. These findings provide a theoretical framework and experimental validation for implementing recycled fine aggregates in sustainable ecological slope protection engineering.

## 1. Introduction

Since the 21st century, accelerated industrialization and urbanization have led to extensive coverage of natural surfaces by impervious materials, severely disrupting regional water circulation systems. Ecological challenges including urban flooding, groundwater depletion, and heat island effects have become increasingly severe [1,2]. The “sponge city” concept emerged to enhance urban capacity for rainwater storage, infiltration, and purification [3,4]. Pervious concrete has become a key paving material due to its continuous pore structure, effectively promoting rainwater infiltration, replenishing groundwater, and reducing surface runoff [5,6].

However, large-scale application of pervious concrete faces an inherent contradiction between strength and permeability. High porosity ensures permeability but weakens paste–aggregate bonding, resulting in compromised mechanical strength [7,8,9]. Researchers have pursued two approaches: material optimization and structural innovation. Material optimization includes supplementary cementitious materials such as fly ash [10], silica fume [11], and metakaolin [12], as well as waste glass powder [13,14], which can enhance concrete strength. Concurrently, recycled fine aggregates from construction waste align with resource utilization strategies while offering environmental and economic benefits [15,16]. Nevertheless, recycled fine aggregates possess inherent limitations. Their high porosity, strong water absorption, and weak interfacial transition zones lead to strength degradation as replacement rates increase [17]. At elevated replacement levels, strength loss becomes particularly pronounced. Their incorporation typically trades strength for marginal permeability gains, exacerbating the strength–permeability dilemma [18,19]. Achieving performance synergy with high recycled aggregate utilization remains a critical challenge.

In structural innovation, double-layer composite pervious concrete demonstrates unique advantages. This configuration comprises a highly permeable upper layer and a high-strength dense lower layer. By leveraging distinct layer characteristics, it offers a pathway to reconcile the strength–permeability contradiction [20]. Recent studies have advanced molding techniques [21], water–cement ratio optimization [22], and aggregate gradation design [23,24], confirming the superior performance of double-layer structures compared to single-layer systems.

Despite these advances, existing research exhibits significant deficiencies. Most studies employ natural aggregates. While recycled aggregates have been explored in single-layer applications, replacement rates typically remain below 40% due to strength loss exceeding 25% at higher levels [17,18,19]. Systematic investigation into high-proportion recycled aggregate utilization through double-layer design while maintaining strength–permeability balance is lacking [20,21]. The paste-to-coarse aggregate ratio, a critical parameter governing paste filling and pore connectivity, has unclear influence mechanisms on double-layer performance. Existing studies examine it coupled with other factors [22,23,24], making independent action patterns difficult to reveal and limiting targeted mix optimization. Furthermore, research focuses primarily on road pavement applications [25]. In slope protection, the regulatory effects of double-layer structures on soil moisture retention and the mechanism of vertical moisture redistribution through interlayer interfaces lack direct verification [26].

To address these gaps, this study adopts a single-factor experimental approach to investigate the influence of recycled fine aggregate replacement rate, water–cement ratio, and paste-to-coarse aggregate ratio on porosity, permeability coefficient, water absorption, and compressive strength of double-layer composite pervious concrete. Optimal mix parameters are determined, and small-scale slope model tests validate the engineering effectiveness in enhancing soil moisture retention. This study first integrates high-proportion recycled fine aggregate utilization with double-layer composite design for slope protection applications, providing theoretical and data support for pervious concrete in slope protection engineering.

## 2. Test Materials and Instruments

P.O 42.5 ordinary Portland cement was employed in this study, with its physicochemical properties presented in Table 1. The admixtures utilized satisfied the requirements specified in the China Standard for Concrete Admixtures (GB 50119-2013 [27]). Tap water was used for mixing.

Additionally, both coarse and fine aggregates used in this study were recycled aggregates derived from construction waste. Two aggregate size ranges, 9–19 mm and 19–26 mm, were obtained through sieving. Their basic physical properties are presented in Table 2. To comprehensively characterize the material properties of recycled aggregates, X-ray fluorescence (XRF) spectroscopy was employed for chemical composition analysis. The results are shown in Table 3.

As shown in Table 3, recycled aggregates exhibit distinct chemical composition compared to natural aggregates. The CaO content is significantly higher (16.8–22.4%), primarily attributed to adhered cement hydration products on the aggregate surface, mainly C-S-H gel and Ca(OH)_2_. Manufactured recycled sand, due to its finer particle size and larger specific surface area, retains more residual cement paste on the surface. Consequently, its CaO content reaches up to 22.4%. Additionally, the loss on ignition (LOI) of recycled aggregates (1.5–2.6%) exceeds that of natural aggregates, reflecting the presence of carbonation products and organic impurities on the surface. These chemical characteristics significantly influence the formation and bonding performance of the interfacial transition zone (ITZ) between recycled aggregates and fresh cement paste.

## 3. Experimental Design and Methods

### 3.1. Design of Pervious Concrete Mixing Ratio

**Figure 1:**
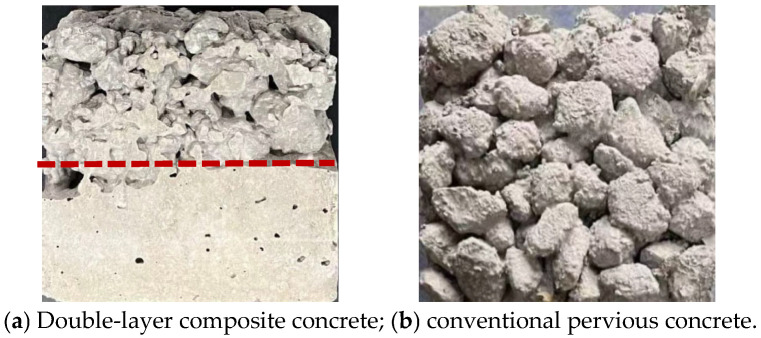
Two types of concrete characteristic surface maps.

As shown in Figure 1a, the concrete developed in this investigation features a composite structure comprising equal proportions (50%/50%) of pervious concrete and conventional concrete. The pervious concrete component prioritizes slow infiltration and high water absorption rather than strength requirements, as detailed in Figure 1b. To achieve this functionality, recycled coarse aggregates in two size fractions (9–19 mm and 19–26 mm) were employed, with a design porosity of 20%.

In the mix proportioning of pervious concrete, the volume equilibrium method was employed for optimization. This method assumes that aggregates, binder, and voids maintain a volume balance within the concrete system, achieving the volume ratio of each component through precise parameter conversion. The volume equilibrium equation is expressed as:

(1) $$V_{a}\;+\;V_{s}\;+\;P\;=\;1$$

where *V*_a_ is the volume fraction of aggregates, *V*_s_ is the volume fraction of binder, and *P* is the volume fraction of voids.

After vibration molding, the aggregates within the pervious concrete achieve a close-packed state. Therefore, the volume and mass of aggregates satisfy the following relationship:

(2) $$V_{a}\;=\;\frac{\alpha_{a}\rho_{a1}m_{a}}{\rho_{a2}}$$

where α is the aggregate modification coefficient (taken as 0.98), $\rho_{a1}$ is the close-packed density of aggregates, ${\;\rho }_{a2}$ is the apparent density of aggregates, and ${\;m}_{a}$ is the mass of aggregates.

In the mix design of pervious concrete, special attention must be paid to the influence of recycled aggregate water absorption on the water–cement ratio. Based on the measured 30 min water absorption rate of recycled aggregates and the design replacement ratio, additional water was preliminarily calculated to compensate for absorption losses. During actual mixing, water content was dynamically adjusted by observing the workability of the mixture to ensure compliance with the effective water–cement ratio design requirements. The volume fractions and mass relationships of the paste components (cement, sand, and water) in pervious concrete satisfy the following:

(3) $$V_{s}\;=\;\frac{m_{c}}{\rho_{c}}\;+\;\frac{m_{s}}{\rho_{s}}\;+\;\frac{m_{w}}{\rho_{w}}$$

(4) $$\frac{m_{s}}{\rho_{s}}=\frac{\alpha_{s}m_{c}}{\rho_{c}}$$

(5) $$m_{r}=(m_{c}+m_{s})\alpha_{r}$$

where *m*_c_, *m*_s_, and *m*_w_ denote the masses of cement, sand, and water, respectively; *ρ*_c_, *ρ*_s_, and *ρ*_w_ denote the densities of cement, sand, and water, respectively; α_s_ is the sand content; *m*_r_ is the mass of water reducer; and α_r_ is the dosage of water reducer.

It should be noted that no water reducer was used in the pervious concrete mix design in this study. The above equations were not fully applied for calculation but are provided to illustrate the basic principles and parameter relationships in the theoretical design.

### 3.2. Specimen Preparation and Molding Process

#### 3.2.1. Preparation of Pervious Concrete Specimens

**Figure 2:**
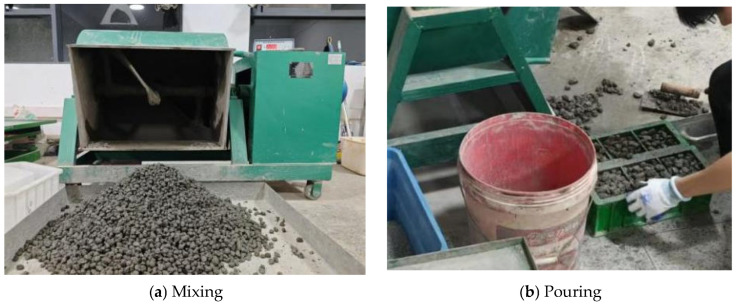
Preparation of concrete specimens.

To eliminate the influence of initial moisture content on mix proportions, recycled fine aggregates were dried at 105 ± 5 °C for 24 h until constant weight before testing. Coarse aggregates were used in air-dried condition with actual moisture content measured for mix adjustment. To address insufficient paste fluidity from low water–cement ratio, a staged batching process was adopted. Cement and recycled fine aggregates were dry-mixed for 30 s, followed by adding 50% mixing water and mixing for 2 min to form uniform mortar. Subsequently, all coarse aggregates, remaining water, and superplasticizer were added gradually. Mixing continued until paste uniformly coated aggregate surface, with total mixing time of at least 3 min. For pervious concrete specimens, material was filled to approximately 20 mm above mold height and vibrated for 10 s. After observing compaction, additional material was added, followed by 5 s vibration until paste appeared on surface. For double-layer specimens, the lower dense layer (approximately 2/3 mold volume) was filled and vibrated for 20–30 s until preliminary compaction. Before initial setting (within 30 min), the upper pervious layer was immediately filled to 20 mm above mold height, with vibration process same as single-layer specimens to ensure tight interlayer bonding. After molding, specimens were sealed with polyethylene film, demolded after 24 h, then cured in standard conditions (temperature 20 ± 2 °C, relative humidity ≥ 95%) for 28 days before testing. The preparation process of the concrete specimens is illustrated in Figure 2.

#### 3.2.2. One-Way Experimental Design

To investigate the effects of recycled fine aggregate replacement rates (0%, 15%, 30%, 45%, 60%) and water–cement ratios (0.27, 0.30, 0.33, 0.36, 0.39) on pervious concrete performance, this study adopted a water–cement ratio design slightly higher than conventional standards. This approach was employed to meet the compositional requirements of composite pervious concrete. The experimental design calculated the combined water and aggregate absorption water as the total water content.

Single-factor tests were first conducted at a water–cement ratio of 0.30. Mix proportions are shown in Table 4. Based on the replacement rate results, a water–cement ratio single-factor test was then performed at 45% replacement rate. The corresponding mix proportions are shown in Table 5.

The mix design for the double-layer composite concrete was based on the experimental results of pervious concrete, with mixture CRS-4 selected as the reference composition. To form the composite structure, the mass of mortar was increased to fill the voids in the lower half of the pervious concrete layer, while maintaining constant coarse aggregate mass, recycled fine aggregate replacement ratio, and water–cement ratio. In the CRS-4 mixture, recycled coarse aggregates accounted for 72% by mass, with a paste-to-coarse aggregate ratio (defined as the mass ratio of cement plus recycled fine aggregate to coarse aggregate) of 0.3. To achieve optimal molding performance for the composite concrete, this paste-to-coarse aggregate ratio was increased accordingly. The specific mix proportions are presented in Table 6.

### 3.3. Test Indicators

**Figure 3:**
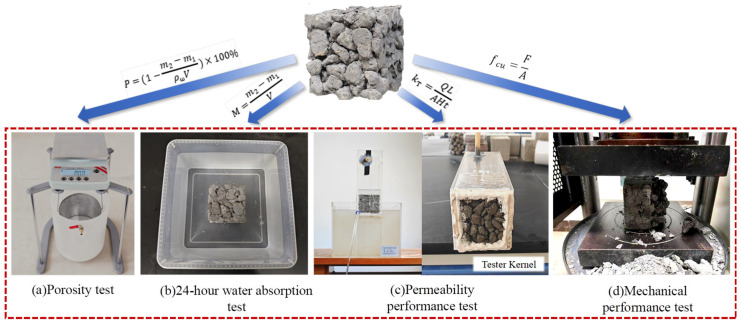
Performance test experiment.

For conventional pervious concrete, strength, permeability coefficient, water absorption, and porosity were measured. For double-layer composite concrete, only strength, water absorption, and porosity were measured due to the absence of vertical through-pores, as shown in Figure 3. Compressive strength testing was conducted using an electro-hydraulic servo compression testing machine with specimen dimensions of 150 mm × 150 mm × 150 mm. The loading rate was controlled at 0.5–0.8 MPa/s according to GB/T 50081-2019 standard [28]. Permeability coefficient testing adopted the constant head permeability method with a head difference of 300 mm and specimen diameter of 100 mm. After water flow stabilized, the permeation flow rate was measured over 60 s, and the permeability coefficient was calculated according to Darcy’s law, following the CJJ/T 135-2009 standard [29]. Porosity testing used the drainage method by measuring the mass difference of specimens in air and water to calculate volumetric porosity. Water absorption testing involved completely immersing specimens in water for 24 h, removing them, wiping off surface moisture, and weighing according to the GB/T 17671-2021 standard [30]. The measurement results for each group were reported as the average of three specimens to ensure data reliability.

## 4. Results and Analyses

### 4.1. Effect of Recycled Aggregate Replacement Ratio

The experimental results of two groups of specimens with different replacement rates are presented in Table 7.

#### 4.1.1. Porosity

**Figure 4:**
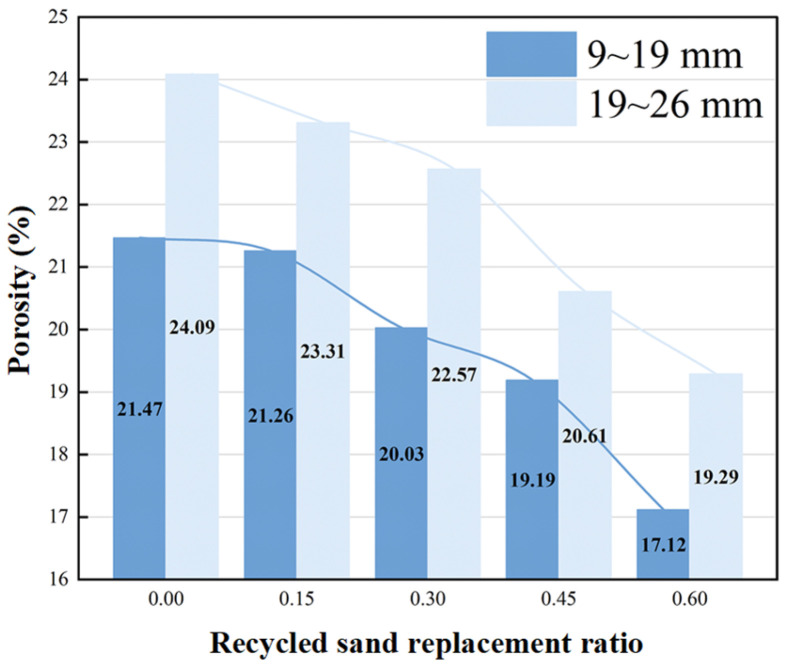
Replacement rate—porosity relationship.

As shown in Figure 4, with the increase in recycled aggregate replacement rate, the porosity of pervious concrete decreases. When the replacement rate increases from 0% to 60%, the porosity of the 19–26 mm aggregate group decreases by 4.80%, which is significantly lower than the 4.35% decrease observed in the 9–19 mm group. This indicates that replacing recycled aggregate with coarser particles can effectively suppress porosity reduction. This phenomenon can be attributed to the angular shape of recycled aggregate, which tends to interlock during compaction, thereby reducing pore spaces. Additionally, the rough surface texture and angular morphology of recycled aggregate also contribute to decreased porosity. Moreover, it is evident that porosity increases with aggregate size, which is consistent with previous findings in the literature.

#### 4.1.2. Permeability Coefficient

**Figure 5:**
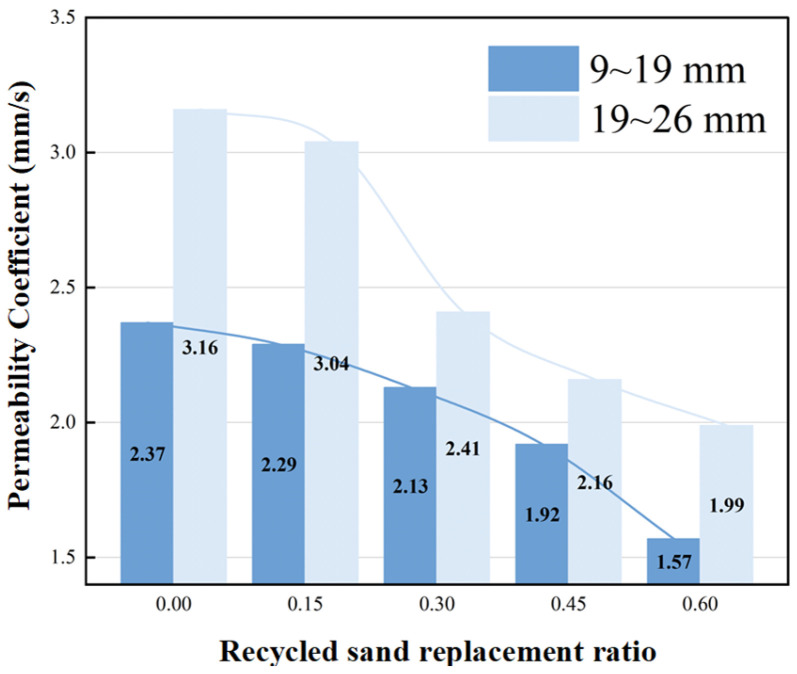
Relationship between replacement rate and permeability coefficient.

As shown in Figure 5, the permeability coefficient is positively correlated with pore connectivity but constrained by the uniformity of paste distribution; it decreases synchronously with the increase in recycled aggregate replacement rate, indicating deterioration of pore connectivity. For the 9–19 mm aggregate group, the permeability coefficient decreases from 2.37 mm/s to 1.57 mm/s, representing a reduction of 33.8%. For the 19–26 mm group, it decreases from 3.16 mm/s to 1.99 mm/s, with a reduction of 37.0%. This phenomenon is primarily attributed to the clogging of interconnected pores by fine particles in the recycled aggregate. It is noteworthy that the permeability coefficient of the 19–26 mm aggregate group consistently remains higher than that of the 9–19 mm group, as its large-pore skeleton structure with open characteristics is more favorable for water infiltration. Moreover, at higher replacement rates, the clogging effect of fine particles in recycled aggregate intensifies.

#### 4.1.3. 24 h Water Absorption Rate

**Figure 6:**
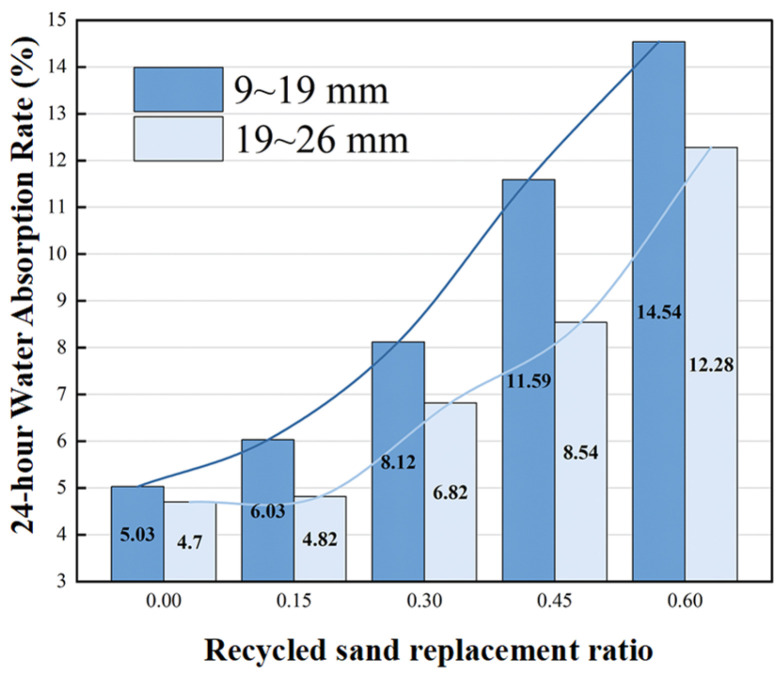
Replacement rate—24 h water absorption relationship.

Water absorption is governed by capillary effects and the inherent water absorption of recycled aggregates. As shown in Figure 6, water absorption increases significantly with recycled sand replacement rate, with the small aggregate group (9–19 mm) showing more pronounced increases. This phenomenon results from three synergistic factors. First, recycled sand absorbs substantial free water during mixing, leaving micropores within hardened aggregates that form dense capillary channels. Second, at high replacement rates (>30%), recycled sand filling reduces pore tortuosity, creating more continuous open water absorption pathways. Third, cement hydration products (C-S-H gel, Ca(OH)_2_) and residual old paste on recycled aggregate surfaces are rich in siloxane groups (Si-O) and hydroxyl groups (-OH). These polar groups form hydrogen bonds with water molecules, imparting hydrophilic characteristics. As recycled sand replacement rate increases, the enrichment of oxygen-containing components such as CaO and Al_2_O_3_ increases hydrophilic active sites, strengthening hydrogen bonding. The high specific surface area of recycled sand amplifies this effect, manifesting as a continuous increase in water absorption. Experimental data show that water absorption of the 9–19 mm aggregate group increased from 5.03% to 14.54% (189% increase), significantly higher than the 19–26 mm group due to smaller pore diameter and stronger capillary effects.

#### 4.1.4. Mechanical Properties

**Figure 7:**
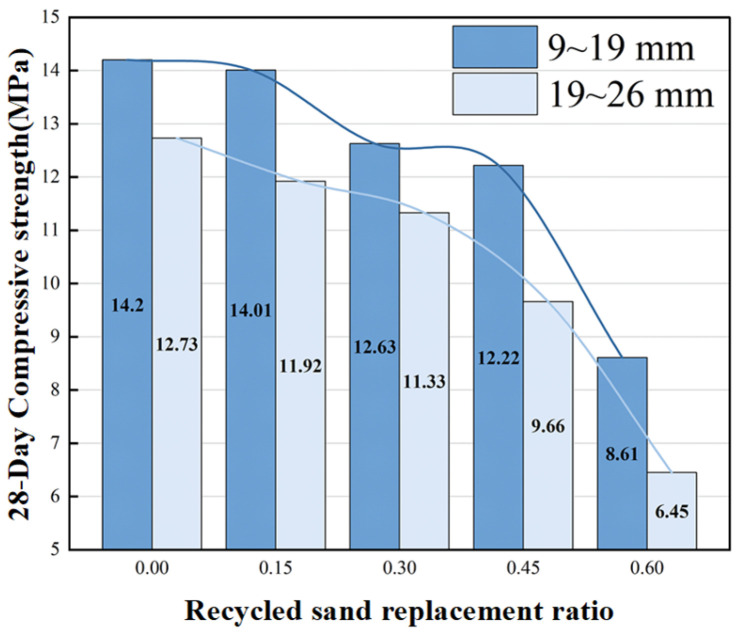
Replacement rate—28 day compressive strength relationship.

As shown in Figure 7, the replacement of cement with recycled sand leads to a reduction in the total amount of cementitious materials, resulting in a continuous decrease in compressive strength with increasing replacement rate. When the replacement rate reaches 60%, the strength of the 9–19 mm aggregate group drops to 8.61 MPa (a reduction of 39.4%), while the 19–26 mm group decreases to 6.45 MPa (a reduction of 49.3%). The strength reduction mainly stems from two aspects. First, recycled sand lacks cementitious activity, leading to reduced hydration products and weakened interfacial transition zone strength. Second, the rough surface of recycled sand with numerous microcracks intensifies interfacial stress concentration, with large-particle-size aggregates experiencing more significant strength loss due to fewer contact points. Furthermore, the high water absorption characteristic of recycled sand reduces the effective water–cement ratio, particularly at replacement rates exceeding 30%, further inhibiting hydration reactions, which is a key factor in strength deterioration.

### 4.2. Effect of Water–Cement Ratio

The water–cement ratio is a key factor affecting the fluidity and ultimate strength of concrete. After determining the appropriate recycled sand replacement rate, it is necessary to establish a suitable water–cement ratio to meet the requirements for molding effect and performance parameters. The test results of various parameters under the influence of the water–cement ratio are shown in Table 8.

#### 4.2.1. Porosity

**Figure 8:**
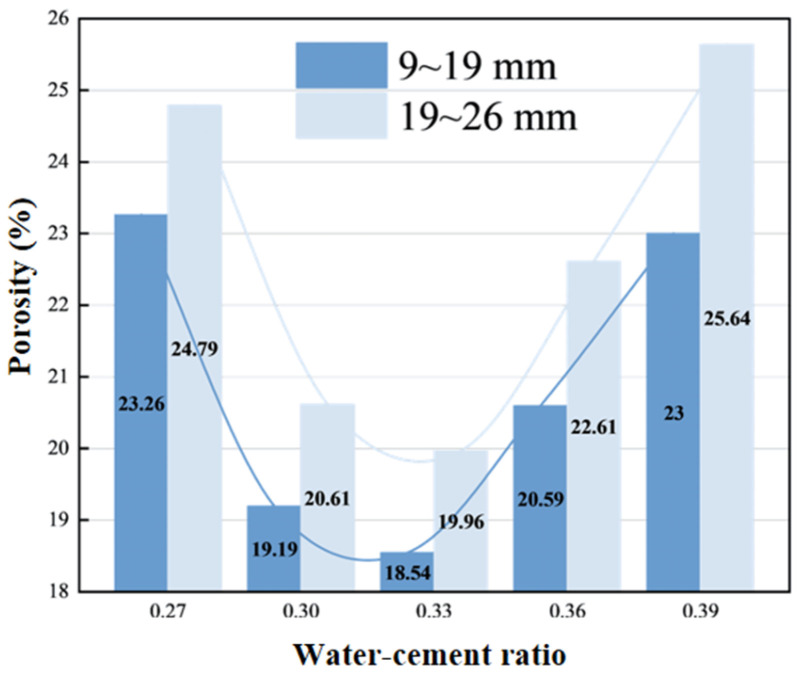
Water–cement ratio—porosity relationship.

As clearly shown in Figure 8, both aggregate groups exhibit the lowest porosity at a water–cement ratio of 0.33, where paste fluidity is optimized to effectively fill the voids between aggregates. When the water–cement ratio decreases (0.27), the viscous paste leads to insufficient coating, increasing porosity to 23.26%. When the water–cement ratio increases (0.39), paste settlement causes loose aggregate packing, resulting in porosity rising back to 23%. The 19–26 mm aggregate group consistently exhibits a higher porosity than the smaller aggregate group due to the low packing density of large-particle-size aggregates, and tends to form more open channels, indicating the decisive influence of aggregate particle size on porosity. Although both excessively high and low water–cement ratios result in high porosity, the pore development under these conditions is unhealthy and unsuitable for pervious concrete design.

#### 4.2.2. Permeability Coefficient

**Figure 9:**
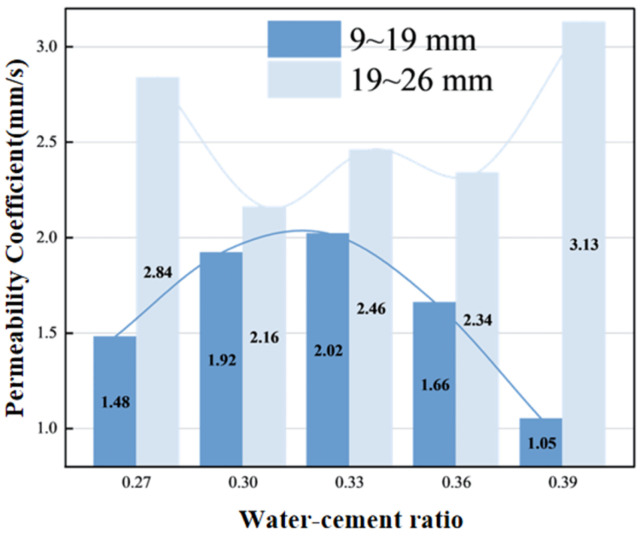
The relationship between water–cement ratio and permeability coefficient.

As shown in Figure 9, aggregate particle size significantly affects the variation in permeability coefficient with water–cement ratio. Large-particle-size aggregates exhibit higher overall permeability coefficients. For small-particle-size aggregates, permeability is low at low water–cement ratios due to viscous paste, poor pore connectivity, and blockage by fine powder. When the water–cement ratio increases to 0.33, improved fluidity promotes an increase in interconnected pores, with the permeability coefficient reaching its peak value. Excessively high water–cement ratios lead to paste settlement that forms closed pores, resulting in decreased permeability. For large-particle-size aggregates, uneven paste coating at low water–cement ratios creates non-uniform pores, yielding higher permeability coefficients. At high water–cement ratios (0.39), the thin paste facilitates the formation of continuous permeable channels, with the permeability coefficient rising against the trend to 3.13 mm/s, demonstrating sensitivity to pore morphology. When the water–cement ratio is between 0.30 and 0.36, moderate paste fluidity and uniform pore distribution lead to stabilized permeability coefficients.

#### 4.2.3. 24 h Water Absorption Rate

**Figure 10:**
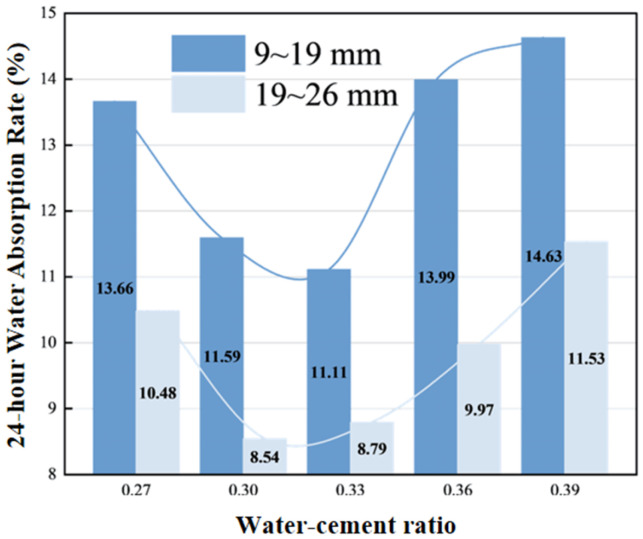
Water–cement ratio—24 h water absorption relationship.

As shown in Figure 10, the 9–19 mm aggregate group exhibits the lowest water absorption at a water–cement ratio of 0.33, primarily because the paste is relatively dense, reducing capillary pores. When the water–cement ratio increases to 0.39, the increase in open pores leads to water absorption rising to 14.63%. The 19–26 mm aggregate group generally shows lower water absorption than the small-particle-size group due to its rapid drainage characteristics and smaller specific surface area. As the water–cement ratio increases from 0.27 to 0.33, both aggregate groups show an overall decreasing trend in water absorption. This phenomenon can be attributed to two factors: On one hand, the micropore water absorption characteristics of recycled aggregates significantly increase the overall water absorption, especially at low water–cement ratios where unhydrated cementitious materials cannot seal the pores in recycled sand. On the other hand, recycled sand cannot effectively coat aggregates under low water–cement ratio conditions, resulting in increased overall porosity of the specimens, which also leads to higher water absorption.

#### 4.2.4. Mechanical Properties

**Figure 11:**
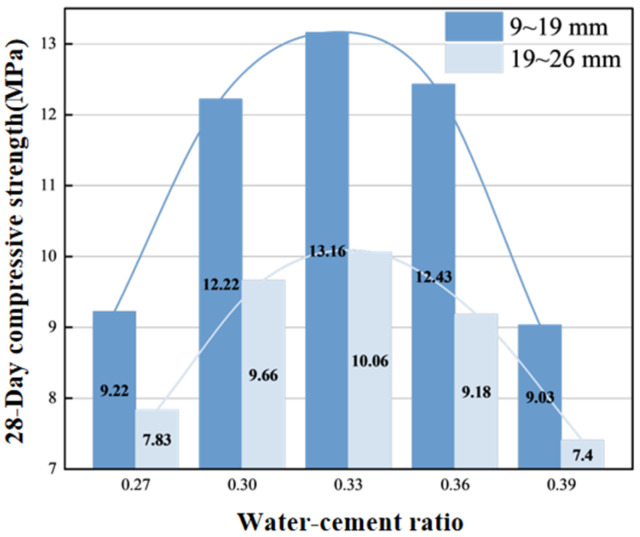
Water–cement ratio—28 day compressive strength relationship.

Figure 11 clearly demonstrates the dual regulation of compressive strength by water–cement ratio and recycled aggregate replacement rate. For the 9–19 mm aggregate group, compressive strength reaches its peak value of 13.16 MPa at a water–cement ratio of 0.33, where paste density and aggregate–paste interfacial bond strength are optimal. The high water absorption of recycled aggregates (5.2%) leads to a reduction in effective water–cement ratio. When the water–cement ratio increases to 0.39, paste coating capacity decreases, and strength drops to 9.03 MPa, representing a 31.5% reduction. The 19–26 mm aggregate group exhibits generally lower compressive strength than the small aggregate group due to weak interfacial transition zones, which is directly related to the stress concentration effect at the large aggregate–paste interface. Furthermore, microcracks and low reactivity of recycled aggregates further exacerbate strength loss, and this effect is more pronounced in the small-particle-size aggregate group.

### 4.3. Effect of Paste-to-Coarse Aggregate Ratio

The paste-to-coarse aggregate ratio is a parameter defined in this study. It represents the mass ratio of paste (cement and fine aggregates) to coarse aggregates in concrete. The paste-to-coarse aggregate ratio was varied to achieve the ideal molding effect of double-layer composite concrete. The experimental results of various performance parameters under the influence of the paste-to-coarse aggregate ratio are shown in Table 9.

#### 4.3.1. Molding Effect

To better describe the molding effect of double-layer composite concrete, the height of the ordinary concrete portion in the specimen is denoted as *h*_1_, and the total height of the specimen is denoted as *h*_2_. A molding index *F* is defined. Equation (6) presents the calculation method for *F*.

(6) $$F=\frac{h_{1}}{h_{2}}$$

**Figure 12:**
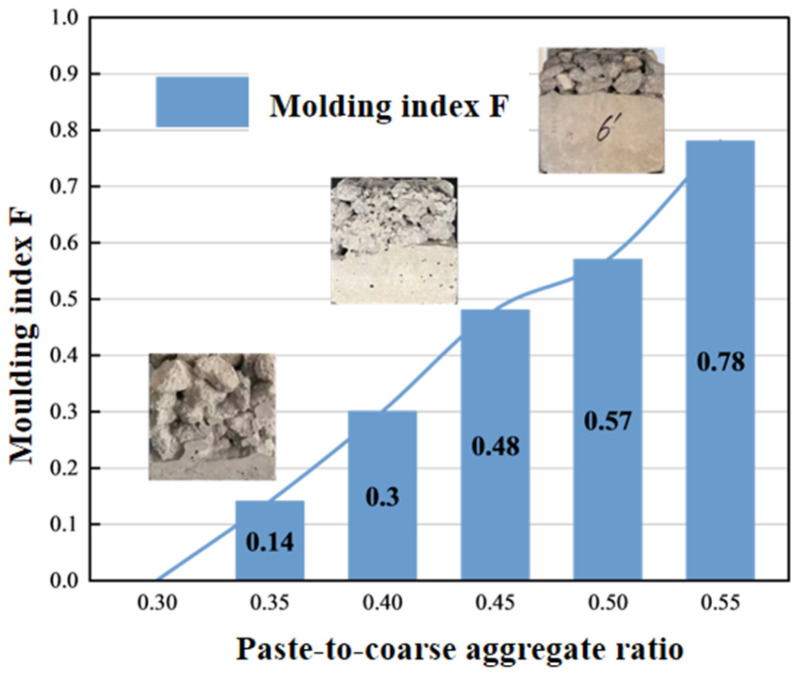
Relationship between paste-to-coarse aggregate ratio and molding index.

When *F* is around 0.5, it can balance both water absorption and permeability and strength. Figure 10 presents the variation in the molding index with increasing paste-to-coarse aggregate ratio. It can be observed that the molding index is positively correlated with the paste-to-coarse aggregate ratio, and the paste-to-coarse aggregate ratio has a significant influence on the final molding effect of concrete. When the paste-to-coarse aggregate ratio is 0.45, the molding index *F* is closest to 0.5. Figure 12 also provides photographs of specimens at paste-to-coarse aggregate ratios of 0.35, 0.45, and 0.55, which intuitively reflect the influence of paste-to-coarse aggregate ratio on the molding effect.

#### 4.3.2. Porosity

**Figure 13:**
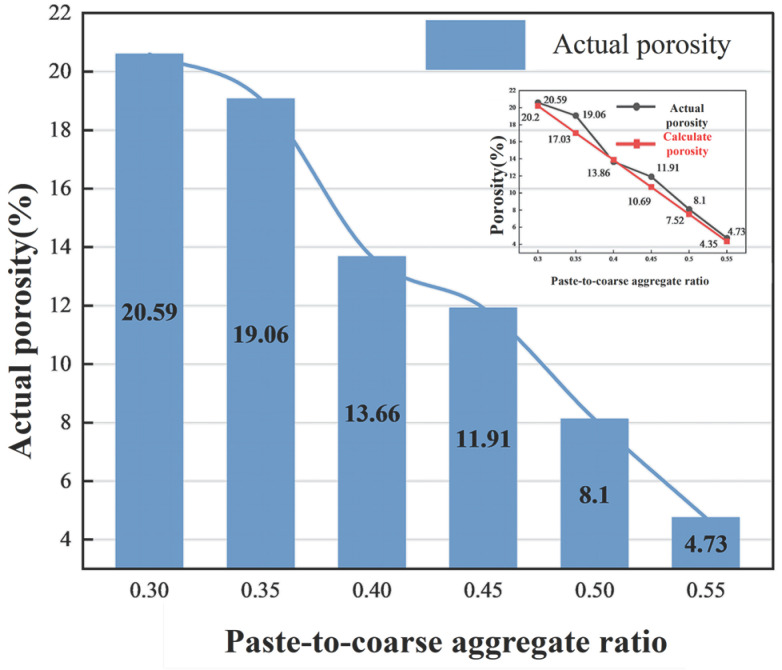
Relationship between paste-to-coarse aggregate ratio and porosity.

The porosity of double-layer composite concrete inevitably shows a decreasing trend with the increase in paste content. When the paste-to-coarse aggregate ratio is relatively small, this decreasing trend is not obvious. As the paste-to-coarse aggregate ratio increases, the voids between coarse aggregates are filled, and the declining trend of porosity intensifies. Figure 13 reflects the relationship between porosity and paste-to-coarse aggregate ratio. When the paste-to-coarse aggregate ratio is 0.3, the actual porosity is basically consistent with the design porosity. However, as the paste-to-coarse aggregate ratio increases, the actual porosity is often greater than the design porosity, which is because the pervious concrete portion in the specimen provides additional pores, having a positive effect on permeability.

#### 4.3.3. 24 h Water Absorption Rate

**Figure 14:**
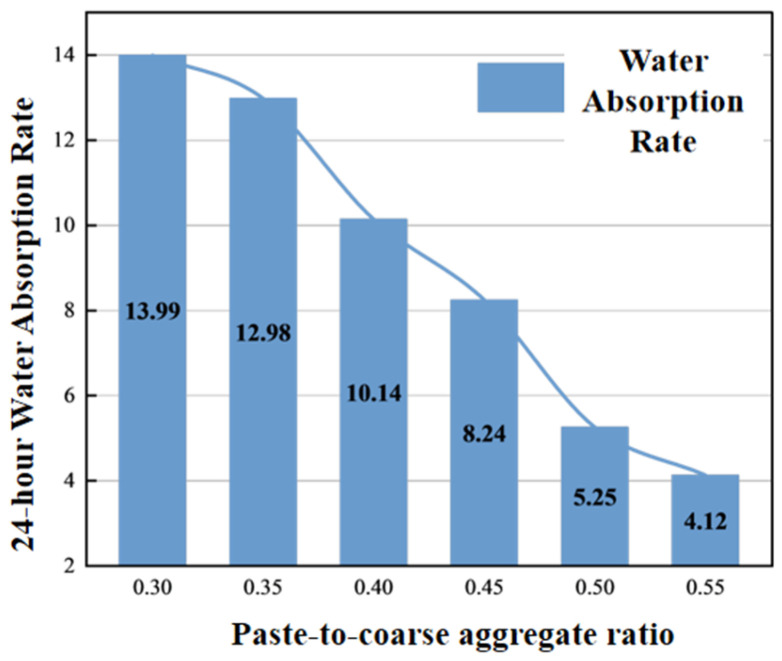
Relationship between paste-to-coarse aggregate ratio and 24 h water absorption.

Water absorption is influenced by both aggregate water absorption and porosity, showing a decreasing trend with increasing paste-to-coarse aggregate ratio. On one hand, increased paste content introduces more recycled fine aggregates, which is beneficial for increasing water absorption. However, water absorption is more significantly affected by porosity. As shown in Figure 14, the variation trend of water absorption is essentially consistent with that of porosity. When the paste-to-coarse aggregate ratio is low, changes in water absorption are not obvious, but as the ratio increases, water absorption decreases sharply.

#### 4.3.4. Mechanical Properties

**Figure 15:**
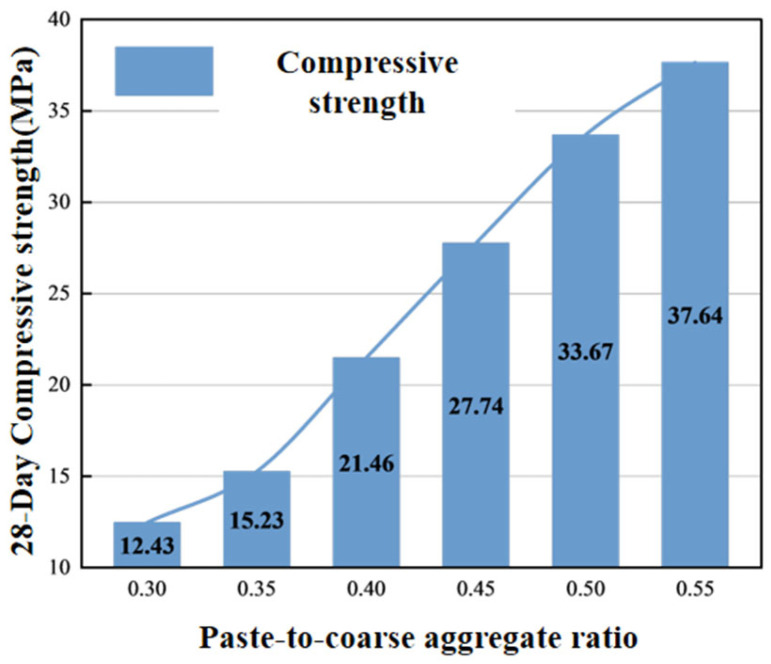
Relationship between paste-to-coarse aggregate ratio and 28 d compressive strength.

Mechanical properties are key factors determining whether double-layer concrete can maintain long-term stability and structural integrity. When the paste-to-coarse aggregate ratio is low, the compressive strength of specimens cannot meet the standard. As the ratio increases, compressive strength gradually improves. The compressive strength of double-layer composite concrete results from the combined effect of the conventional concrete portion and the pervious concrete portion. Comparing Figure 12 and Figure 15, it can be seen that the molding index and compressive strength show high correlation. There is a clear positive correlation between compressive strength and paste-to-coarse aggregate ratio. The compressive strength at a paste-to-coarse aggregate ratio of 0.45 can fully meet the requirements for concrete use.

## 5. Water Retention Evaluation

### 5.1. Slope Protection Block Design

**Figure 16:**
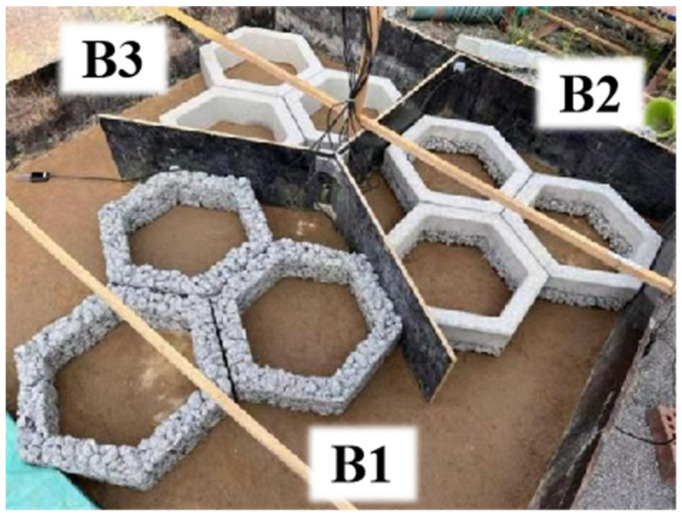
Three types of concrete blocks.

Three different types of slope protection blocks were designed: pervious concrete (B1), composite double-layer concrete (B2), and conventional concrete (B3), as shown in Figure 16. The blocks are regular hexagonal in shape, with an outer side length of 30 cm, inner side length of 20 cm, and thickness of 6 cm. The height of each block is controlled at 15 cm. The pervious concrete blocks use the mix proportion from WCR-4, the double-layer composite concrete blocks adopt the mix proportion from group DC-3, and the conventional concrete blocks use the standard mix proportion for C25 concrete.

### 5.2. Model Test Design

**Figure 17:**
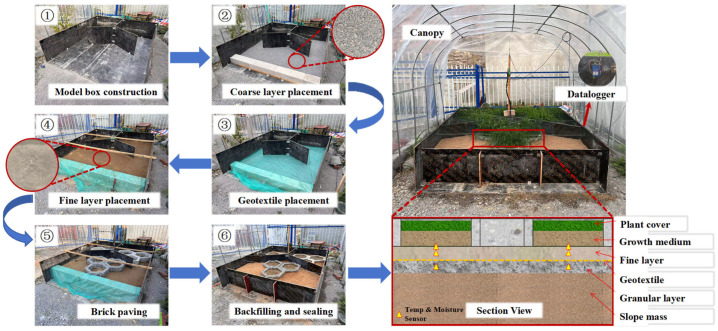
Model construction process and on-site layout.

The experimental model was designed based on an actual ecological protection system for a waterway slope, with a full-scale model constructed. The fine-grained layer used waste mud generated during construction waste resource utilization. This mud belongs to silty soil with a particle size less than 0.075 mm after sieving. By controlling its compaction degree, the fine-grained layer forms a small pore structure conducive to capillary action. The coarse-grained layer also used recycled gravel with a particle size ranging from 2 to 20 mm, forming a large-pore drainage layer. Geotextile was laid between the fine-grained and coarse-grained layers as a transition layer to avoid capillary breakage caused by abrupt particle size changes. The entire model framework was constructed with wooden boards connected by self-tapping screws and metal angle brackets. To better reflect the water retention performance of different blocks in this design, the entire model slope was set horizontally. The model construction process is shown in Figure 17.

To verify the influence of different block types on moisture content in various soil layers of the slope, three block schemes were set up for comparison in the slope model test: pervious concrete blocks (B1), double-layer composite concrete blocks (B2), and conventional concrete blocks (B3). EC-5 soil moisture sensors (Decagon Devices, Pullman, WA, USA, measurement range 0–100% volumetric water content, accuracy ±3%, operating temperature 0–60 °C) were used for moisture monitoring. One sensor was embedded in each of the planting layer, fine-grained layer, and coarse-grained layer for each group, totaling three sensors per group. Data acquisition interval was 30 min with a monitoring period of 15 days to reflect the dynamic variation patterns of moisture content in each soil layer in real time.

### 5.3. Soil Water Retention Analysis

**Figure 18:**
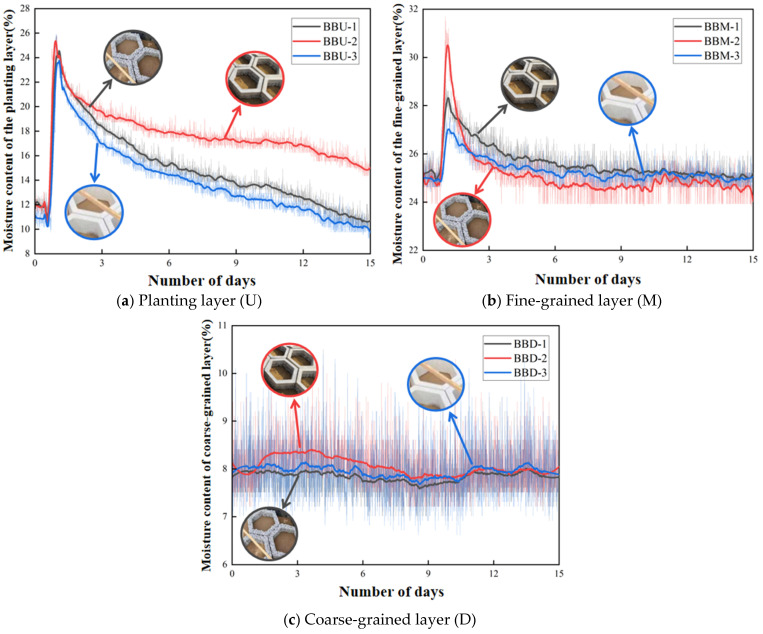
Changes in moisture content of bare bricks in the same soil layer.

After completing the monitoring, graphs showing the variation in moisture content over time for each soil layer were plotted, as shown in Figure 18. For the pervious concrete blocks (B1), sensors in the planting layer, fine-grained layer, and coarse-grained layer are designated as BBU-1, BBM-1, and BBD-1, respectively. For the double-layer composite concrete blocks (B2), sensors in the planting layer, fine-grained layer, and coarse-grained layer are designated as BBU-2, BBM-2, and BBD-2, respectively. For the conventional concrete blocks (B3), sensors in the planting layer, fine-grained layer, and coarse-grained layer are designated as BBU-3, BBM-3, and BBD-3, respectively. The values monitored by soil moisture sensors fluctuate, primarily due to thermal expansion and contraction effects and sensor temperature drift. Soil particles and pores expand or contract with temperature changes, altering moisture distribution and electrical conductivity. The sensitivity of electronic components is also affected by temperature, leading to some degree of reading drift. To better reflect the trends in moisture content changes across soil layers, the original experimental data are plotted with semi-transparent treatment, while the fitted curve trends are displayed with enhanced depth.

As shown in Figure 18, the three block types exhibit distinct moisture retention behaviors across soil layers. In the planting layer, all blocks show rapid initial moisture decline, but the double-layer composite blocks (BBU-2) achieve optimal long-term retention by combining the pervious layer’s absorption capacity with the dense layer’s seepage prevention. While pervious concrete blocks (BBU-1) absorb water effectively, surface evaporation is rapid; conventional blocks (BBU-3) decline fastest due to poor absorption and lack of retention structure—despite similar curve patterns between BBU-1 and BBU-3, their mechanisms differ fundamentally. In the fine-grained layer, block structure significantly influences early-stage moisture migration, with the double-layer composite structure slowing infiltration such that BBM-2 reaches the highest post-irrigation moisture content. BBM-1’s interconnected pores promote excessive lateral movement, intensifying evaporation while slowing infiltration—still achieving favorable moisture increase with better short-term retention than BBM-3, whose minimal fluctuation suggests its non-porous structure limits evaporation but fails to slow infiltration. The coarse-grained layer exhibits minimal overall fluctuation with slight decline followed by slight increase, with BBD-2 showing a notable sharp spike after irrigation that, combined with BBM-2 observations, suggests capillary breakthrough at the fine–coarse interface causes water redistribution. Long-term results demonstrate BBU-2’s clear advantage in maintaining overall moisture balance across soil layers.

## 6. Conclusions

This study analyzed the variation in pervious concrete material properties with mortar replacement ratio and water–cement ratio, as well as the influence of paste-to-coarse aggregate ratio on double-layer composite concrete, ultimately determining the optimal design parameters as: water–cement ratio of 0.36, mortar replacement ratio of 45%, and paste-to-coarse aggregate ratio of 0.45. The main conclusions are as follows:

(1) Porosity is governed by the synergistic effect of recycled sand filling and aggregate particle size, where increased recycled sand leads to greater paste volume that fills voids between coarse aggregates, while both low and high water–cement ratios affect pore development through insufficient paste coating and paste settlement respectively.

(2) Water absorption is primarily attributed to the absorbency of recycled materials and capillary effects in pores, as the addition of recycled fine aggregate significantly enhances water absorption due to both its inherent high absorbency and rough surface that intensifies capillary water uptake.

(3) Compressive strength exhibits dominant mechanisms of cementitious dilution and interfacial weakening, being jointly regulated by water–cement ratio and aggregate particle size in pervious concrete—high water–cement ratios cause sharp strength deterioration and recycled sand replacement ratio should not exceed 0.45, while in double-layer composite concrete, excessively low paste-to-coarse aggregate ratios fail to meet code requirements.

(4) Permeability coefficient demonstrates a balance between pore connectivity and recycled sand clogging, increasing when water–cement ratio is either too high or too low and when recycled sand replacement ratio is low, with large-pore frameworks exhibiting superior connectivity and permeability compared to small-pore structures.

(5) Composite concrete blocks demonstrate relatively stable moisture content changes and minimal fluctuation ranges across all soil layers, indicating structural stability suitable for slope protection projects requiring moisture stability.

This study has certain limitations. It currently focuses on mix optimization of recycled aggregates and macroscopic performance evaluation at 28-day age, without conducting durability tests such as freeze–thaw resistance, abrasion resistance, and clogging resistance. Additionally, the recycled aggregates used are from a single source, while aggregates from different sources vary in adhered mortar content and impurity levels. Future research should incorporate multi-source aggregate comparisons to enhance the generalizability of conclusions. Furthermore, the interfacial characteristics and time-dependent effects of the double-layer structure have not been systematically investigated. The shrinkage cracking behavior of recycled aggregate concrete under restrained conditions [31] and the interfacial stress caused by shrinkage differences between upper and lower layers in double-layer structures [32] have significant impacts on long-term service performance. Interfacial transition zone characteristics and time-dependent effects such as shrinkage and creep represent important directions for future research.

## Figures and Tables

**Table 1:** Cement physical and chemical indicators.

Apparent Density	Specific Surface Area	Initial Setting Time	Final Setting Time	Chemical Composition (Mass Fraction, %)					
/(kg/m^3^)	/(m^2^/kg)	/min	/min	SiO_2_	Fe_2_O_3_	Al_2_O_3_	CaO	MgO	SO_3_
3100	340	100	240	21.3	3.1	5.2	62.6	2.8	2.6

**Table 2:** Basic physical properties of relevant aggregates.

Aggregate Type	Apparent Density/(kg/m^3^)	Bulk Density/(kg/m^3^)	Porosity/%	Water Absorption/%	Crushing Index/%	Fineness Modulus
Recycled coarse aggregate 9–19 mm	2350	1550	34	2.2	23	/
Recycled coarse aggregate 19–26 mm	2250	1650	41	2.4	28	/
Machine-manufactured recycled fine aggregate	2550	1650	/	5.2	22	2.8

**Table 3:** Chemical composition of aggregates.

Aggregate Type	SiO_2_/%	Al_2_O_3_/%	Fe_2_O_3_/%	CaO/%	MgO/%	SO_3_/%	Loss on Ignition/%
Recycled coarse aggregate 9–19 mm	58.3	12.8	4.2	18.6	2.4	1.2	2.5
Recycled coarse aggregate 19–26 mm	61.2	11.5	3.9	16.8	2.6	1.4	2.6
Machine-manufactured recycled fine aggregate	54.7	13.6	4.8	22.4	2.1	0.9	1.5

**Table 4:** Permeable concrete mix proportion under the influence of replacement rate.

Groups	Type of Coarse Aggregate	Water–Cement Ratio	Replacement Rate/%	Coarse Aggregate/kg	Fine Aggregate/kg	Cement/kg	Total Water/kg	Mixing Water/kg
CSR-1	9–19 mm	0.3	0	1600	0	480	180	144
CSR-2	9–19 mm	0.3	0.15	1625	74	413	163	125
CSR-3	9–19 mm	0.3	0.3	1651	149	348	149	103
CSR-4	9–19 mm	0.3	0.45	1680	226	278	132	84
CSR-5	9–19 mm	0.3	0.6	1711	307	204	115	60
CSR-6	19–26 mm	0.3	0	1594	0	480	182	144
CSR-7	19–26 mm	0.3	0.15	1622	74	413	166	125
CSR-8	19–26 mm	0.3	0.3	1649	149	346	151	103
CSR-9	19–26 mm	0.3	0.45	1680	226	276	137	84
CSR-10	19–26 mm	0.3	0.6	1706	307	204	118	62

**Table 5:** The mix proportion of permeable concrete under the influence of water–cement ratio.

Groups	Type of Coarse Aggregate	Water–Cement Ratio	Replacement Rate/%	Coarse Aggregate/kg	Fine Aggregate/kg	Cement/kg	Total Water/kg	Mixing Water/kg
WCR-1	9–19 mm	0.27	0.45	1692	228	278	125	74
WCR-2	9–19 mm	0.3	0.45	1680	226	278	132	84
WCR-3	9–19 mm	0.33	0.45	1670	226	276	139	89
WCR-4	9–19 mm	0.36	0.45	1656	223	274	146	98
WCR-5	9–19 mm	0.39	0.45	1646	223	271	154	106
WCR-6	19–26 mm	0.27	0.45	1690	228	278	127	74
WCR-7	19–26 mm	0.3	0.45	1680	228	276	134	84
WCR-8	19–26 mm	0.33	0.45	1666	223	276	142	91
WCR-9	19–26 mm	0.36	0.45	1656	223	274	149	98
WCR-10	19–26 mm	0.39	0.45	1644	223	271	158	106

**Table 6:** Mix proportion of double-layer composite concrete.

Groups	Type of Coarse Aggregate	Water–Cement Ratio	Replacement Rate/%	Coarse Aggregate/kg	Fine Aggregate/kg	Cement/kg	Total Water/kg	Mixing Water/kg
DC-0	9–19 mm	0.36	0.45	0.3	1656	223	274	146
DC-1	9–19 mm	0.36	0.45	0.35	1656	261	319	165
DC-2	9–19 mm	0.36	0.45	0.4	1656	298	364	183
DC-3	9–19 mm	0.36	0.45	0.45	1656	335	410	201
DC-4	9–19 mm	0.36	0.45	0.5	1656	373	455	220
DC-5	9–19 mm	0.36	0.45	0.55	1656	410	501	238

**Table 7:** Experimental results under the influence of replacement rate.

Groups	Type of Coarse Aggregate	Water–Cement Ratio	Replacement Rate/%	Porosity/%	Compressive Strength/MPa	Permeability Coefficient/mm/s	Water Absorption Rate/%
CSR-1	9–19 mm	0.30	0.00	21.47	14.20	2.37	5.03
CSR-2	9–19 mm	0.30	0.15	21.26	14.01	2.29	6.03
CSR-3	9–19 mm	0.30	0.30	20.03	12.63	2.13	8.12
CSR-4	9–19 mm	0.30	0.45	19.19	12.22	1.92	11.59
CSR-5	9–19 mm	0.30	0.60	17.12	8.61	1.57	14.54
CSR-6	19–26 mm	0.30	0.00	24.09	12.73	3.16	4.70
CSR-7	19–26 mm	0.30	0.15	23.31	11.92	3.04	4.82
CSR-8	19–26 mm	0.30	0.30	22.57	11.33	2.41	6.82
CSR-9	19–26 mm	0.30	0.45	20.61	9.66	2.16	8.54
CSR-10	19–26 mm	0.30	0.60	19.29	6.45	1.99	12.28

**Table 8:** Test results under the influence of water–cement ratio.

Groups	Type of Coarse Aggregate	Water–Cement Ratio	Replacement Rate/%	Porosity/%	Compressive Strength/MPa	Permeability Coefficient/mm/s	Water Absorption Rate/%
WCR-1	9–19 mm	0.27	0.45	23.26	9.22	1.48	13.66
WCR-2	9–19 mm	0.3	0.45	19.19	12.22	1.92	11.59
WCR-3	9–19 mm	0.33	0.45	18.54	13.16	2.02	11.11
WCR-4	9–19 mm	0.36	0.45	20.59	12.43	1.66	13.99
WCR-5	9–19 mm	0.39	0.45	23.00	9.03	1.05	14.63
WCR-6	19–26 mm	0.27	0.45	24.79	7.83	2.84	10.48
WCR-7	19–26 mm	0.3	0.45	20.61	9.66	2.16	8.54
WCR-8	19–26 mm	0.33	0.45	19.96	10.06	2.46	8.79
WCR-9	19–26 mm	0.36	0.45	22.61	9.18	2.34	9.97
WCR-10	19–26 mm	0.39	0.45	25.64	7.40	3.13	11.53

**Table 9:** Test results of double-layer composite concrete.

Groups	Type of Coarse Aggregate	Water–Cement Ratio	Replacement Rate/%	Paste-to-Coarse Aggregate Ratio	Proportion of Pervious Concrete Portion	Porosity/%	Compressive Strength/MPa	Water Absorption Rate/%
DC-0	9–19 mm	0.36	0.45	0.3	1.00	20.59	12.43	13.99
DC-1	9–19 mm	0.36	0.45	0.35	0.86	19.06	15.23	12.98
DC-2	9–19 mm	0.36	0.45	0.4	0.70	13.66	21.46	10.14
DC-3	9–19 mm	0.36	0.45	0.45	0.52	11.91	27.74	8.24
DC-4	9–19 mm	0.36	0.45	0.5	0.43	8.10	33.67	5.25
DC-5	9–19 mm	0.36	0.45	0.55	0.22	4.73	37.64	4.12

## Data Availability

The original contributions presented in this study are included in the article. Further inquiries can be directed to the corresponding author.
